# Study to assess whether staff are able to accurately assess sleep quality and quantity in intensive care patients

**DOI:** 10.1186/cc13601

**Published:** 2014-03-17

**Authors:** J Patel, J Baldwin, P Bunting, S Laha

**Affiliations:** 1 Royal Preston Hospital, Manchester, UK

## Introduction

Sleep deprivation is recognised as an important cause of morbidity after ICU admission, but most centres do not routinely assess their patients' sleep. Considering the invasive nature and costs associated with objective sleep measurements, they are unsuitable for routine use. Subjective measurements offer an easy-to-use and economical alternative, the most well validated of which is the Richards-Campbell Sleep Questionnaire (RCSQ). This can be used to derive an accurate estimation of the sleep efficiency index (SEI), a well-validated measure of sleep. However, there are several concerns regarding patients reporting their sleep quality and quantity using these questionnaires [1]. Additionally, they cannot be used to assess sleep in sedated or delirious patients. It has been suggested that one way to bypass the drawbacks of patients assessing their own sleep would be to utilise nursing staff [1]. Previous smaller scare studies have since agreed with this suggestion [2]. This study aimed to assess whether staff were able to use the RCSQ to accurately assess their patients' sleep.

## Methods

Fifty-nine patients consented to complete the RCSQ for each night of their ICU admission. Alongside this, the nurses who had cared for these patients were asked to assess their patients' sleep using the RCSQ. These were then matched with their patients' responses. The Bland-Altman method was applied to assess for agreement between patient and nurse SEIs in order to reveal whether nurses could accurately estimate their patients' night sleep. Additionally, Cronbach's alpha was derived to assess for internal consistency. Ethical approval was gained prior to the start of the study.

## Results

**Figure 1:**
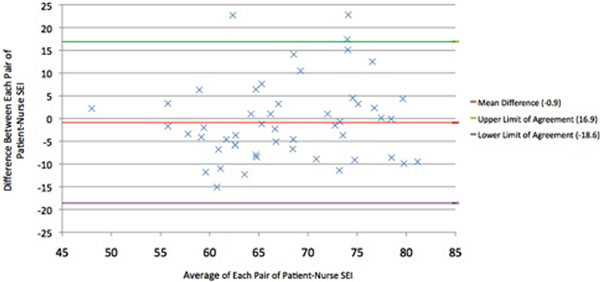


A total of 126 pairs or RCSQs were gathered. The mean difference between nurse and patient SEIs was -0.9, suggesting there is no significant trend regarding nurses overestimating or underestimating patients' SEIs. In total, 94.2% of nurses' estimations fell within the limits of agreement. The variability of the differences was consistent across the range of averages. Cronbach's alpha was 0.63 between nurse and patient scores, suggesting questionable reliability between the RCSQ pairs. See Figure 1.

## Conclusion

The data gathered here demonstrate that nurses are not able to accurately estimate their patients sleep using the RCSQ, and hence alternative methods of sleep monitoring should be developed for routine use.
